# Rheological Properties and Melt Spinning Application of Controlled-Rheology Polypropylenes via Pilot-Scale Reactive Extrusion

**DOI:** 10.3390/polym14153226

**Published:** 2022-08-08

**Authors:** Ho Suk Ji, Geunyeop Park, Hyun Wook Jung

**Affiliations:** 1 Department of Chemical and Biological Engineering, Korea University, Seoul 02841, Korea; 2 Chemical TS&D Planning Team, S-OIL Corporation, S-OIL TS&D Center, Seoul 07793, Korea

**Keywords:** controlled-rheology polypropylene, peroxide-initiated degradation, pilot-scale reactive extrusion, rheological properties, melt spinning process

## Abstract

Based on pilot-scale twin-screw reactive extrusion, the structural and rheological properties of controlled-rheology polypropylenes (CR-PPs) are investigated, where the effects of peroxide content and extrusion conditions such as screw configuration, extrusion temperature, and screw speed are prioritized. The active chain cleavage reaction by a small peroxide content of less than 600 ppm inside the extruder gradually increases the melt index and narrows the molecular weight distribution of CR-PPs, thereby affording favorable properties that are applicable to the fiber spinning process. The mechanical properties of CR-PPs are slightly degraded owing to the generation of unsaturated chain ends during the reactive extrusion, which suppresses crystal growth. Under all extrusion conditions, the chain scission and thermal degradation of polypropylene samples occur actively in the harsh twin-screw extruder compared with those in the mild twin-screw extruder. Finally, it is confirmed that CR-PPs can be suitably applied to the melt-spinning process for staple fiber production, thereby guaranteeing a more stable spinning process window against draw resonance instability.

## 1. Introduction

Polypropylene (PP), as one of the most versatile thermoplastics, has a wide range of uses, including plastic packaging films, membranes, textiles, and high precision plastic parts. It is well established owing to its excellent mechanical properties and chemical resistance at reasonable prices. Considering their outstanding processability, many versatile PP products have been manufactured using various polymer processing methods, such as injection molding, extrusion, and thermoforming [1]. Among the many applications of fiber production, the molecular weight distribution (MWD) of PP should be the narrowest possible to ensure good stretchability and spinnability [2]. The development of target-oriented catalysts has allowed the narrow MWD of PP to be adjusted during the polymerization process. Recently, the role of controlled rheology in upgrading the physical properties of PP has become more important as it allows additives (e.g., peroxide) to be introduced easily in the polymer extrusion stage, compared with catalyst modification [3,4,5,6,7].

Controlled rheology is a method for regulating the melt index (MI) of polymers by incorporating various types of peroxides for processability and narrow MWD. During the extrusion process, peroxides are pyrolyzed into peroxyl radicals, which can transform polymers into low-molecular-weight ones by breaking the polymer backbone through chain scission [8,9,10,11,12,13,14,15]. Relevant studies have been conducted to elucidate the degradation of polymers during extrusion. Because such a peroxide-induced chain scission reaction proceeds naturally throughout the extrusion process, structural changes in the polymer are indispensable in controlled rheology. Krell et al. [9] and Pabedinskas et al. [10] developed theoretical models, including basic chain cleavage reactions using peroxyl radicals, and experimentally verified changes in molecular weights. Lazar et al. [14] discovered that PP degradation not only reduced its molecular weight, but also generated unsaturated C = C or C = O double bonds.

In addition, many studies have been carried out regarding the changes in various properties of PPs due to peroxide-induced chain scission [16,17,18,19,20,21,22]. Rocha et al. [16] observed irregular melting temperatures due to the development of a non-uniform chain structure in extruded PP products as the PP decomposition proceeded gradually. Azizi and Ghasemi [17] and Azizi et al. [18] showed that the complex viscosity of controlled-rheology polypropylenes (CR-PPs) remained constant up to 10 rad/s and then decreased monotonically above that frequency as the peroxide content increased. Tzoganakis et al. [19] and Baik and Tzoganakis [20] discovered that the extrudate swelling of CR-PPs decreased with increasing peroxide content and extrusion speed. In addition, no melt fracture defects occurred, and the degree of extrudate deformation decreased as the shear rate increased for certain material and processing conditions.

Scrutinizing the effect of process conditions during reactive extrusion is crucial when adjusting the properties of CR-PPs [23,24,25,26,27]. Ryu et al. [23] investigated the effect of process temperature on the extrusion of CR-PPs. The decomposition degree of CR-PPs with 0.1 wt% peroxide did not change significantly in the range of 190 to 210 °C, although it increased remarkably at temperatures exceeding 220 °C, resulting in a greater amount of low-molecular-weight PPs. Machado et al. [24] and Berzin et al. [25] demonstrated that the screw combination in a twin-screw extruder promoted the rapid degradation and melting of CR-PPs. Tzoganakis et al. [26] reported that an increase in peroxide content widened the residence time distribution (RTD) owing to reduced melt viscosity in a single-screw extruder. In addition, the increased extrusion temperature resulted in a narrower RTD, whereas the screw speed did not affect the RTD. In addition to CR-PPs, the controlled rheology strategy of ethylene–propylene copolymers and PP/PE blends has been extensively investigated [28,29,30,31,32,33,34]. Unlike in the case of isotactic PPs, the viscosity levels of the other polymeric systems mentioned above were improved as the peroxide content increased, owing to crosslinking [35].

To date, most studies regarding CR-PPs have primarily focused on degradation and rheological changes relative to peroxide content, which were observed using lab-scale mixers or extruders. However, pilot-scale production presents significant challenges such as scale-up development, stable long-term physical properties, and process stability that do not occur in lab-scale approaches. Therefore, for industrially manufacturing high-quality fibers or spunbonds, a controlled rheology strategy for the pilot-scale production of PP should be established. 

In this study, a pilot-scale extrusion process and controlled rheology were combined to account for structural and rheological changes in PP by controlling the peroxide content and processing conditions such as the screw speed and extrusion temperature. Two types of pilot-scale (mild and harsh) twin-screw configurations with an L/D exceeding 40 were adopted. Furthermore, the peroxide remaining inside the extruder after extrusion was considered while changing the extrusion temperature and screw speed. CR-PPs can be stabilized by changing the temperature and pressure inside the extruder and by verifying the MI value. Finally, the applicability of CR-PPs in the melt-spinning process was substantiated by comparing the melt tension and draw resonance instability occurring in the spinline with those of PPs commercialized for staple fibers.

## 2. Experiment

### 2.1. Twin-Screw Extrusion of PP Resins

#### 2.1.1. Twin-Screw Extruders with Different Screw Configurations

**Figure 1:**
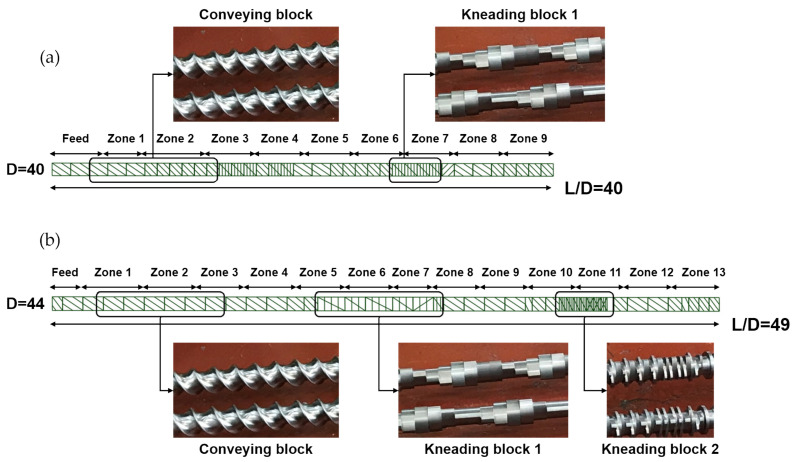
Schematic configurations of (**a**) mild and (**b**) harsh screws in twin-screw extruders.

Two pilot-scale twin-screw extruders with different screw configurations were incorporated to realize peroxide-initiated reactions during PP extrusion through the sequential steps of conveying, melting, mixing, and homogeneous discharging. The twin screws present full intermeshing and co-rotating features but exhibit different shapes and configurations in terms of their elements. Detailed structural specifications of the twin screws are shown in Figure 1. The first twin-screw configuration (Figure 1a), with a screw diameter (D) of 40 mm and an aspect ratio of L/D = 40 (L is the screw length), includes normal conveying and kneading blocks, which is used for general polymer production purposes (at relatively low shear conditions). The conveying block plays a main role in transporting a polymer melt through weak or mild mixing. The kneading block is used to distribute and disperse a polymer resin by mixing it rather strongly. The second twin-screw (Figure 1b), with D of 44 mm and L/D = 49, which is specifically used for glass-fiber compounding (at relatively high shear conditions), comprises additional harsh kneading blocks for intense distributive mixing. Kneading block 2, which has a shorter pitch compared to kneading block 1, provides stronger distribution and dispersion. To distinguish the two twin-screw configurations with different L/D values, screw diameters, and kneading blocks, the former was named “mild” and the latter “harsh”.

In the extrusion processing of PP polymers through mild and harsh twin-screw configurations, three process parameters (i.e., the peroxide content, the screw speed, and the extrusion temperature) were mainly changed in the consideration of PP intrinsic properties to manufacture extruded CR-PPs. The screw speed was set to 200 and 250 rpm and a vacuum pump was installed in the extruder to remove volatile materials. The PP extrudate strands at the die exit were cooled rapidly in a long water bath and pelletized. Peroxides from 0 to 600 ppm were mixed with the PP resin before extrusion. The temperature in each screw zone specified in Figure 1 was set in Table 1 for mild and harsh extrusion conditions. The temperature condition in the last screw zones, where a polymer melt exits the extruder, was named set temperature. 

#### 2.1.2. Materials

For reactive extrusion experiments based on controlled rheology, neat PP with an MI of 4 manufactured by S-Oil (HD500, Seoul, Korea, density (ρ) = 0.743 g/mL, number-average molecular weight ($M_{n}$) = 110,000, and weight-average molecular weight ($M_{w}$) = 410,000) was prepared as a base polymer. A peroxide masterbatch, whose base carrier was homo PP (2.5-dimethyl 2.5-di(tert-butylperoxy) hexane (DTBPH), Copolyman Co., Seoul, Korea), was introduced to improve the processability of PP. The $M_{n}$, $M_{w}$, and MWD of CR-PPs produced via mild and harsh twin-screw extruders were measured using gel permeation chromatography (8312GPC-HT, Tosoh, Tokyo, Japan) to identify the chain scission of PP with respect to the peroxide content. Carrier solvent o-dichlorobenzene at a temperature of 140 °C with flow rate of 1 mL/min and pressure of 25 bar was used.

To demonstrate the effectiveness of controlled rheology on the spinnability of the melt-spinning process, peroxide-treated CR-PP (with a peroxide content of 200 ppm) was compared with other homo PP resins commercialized for staple fiber application. Three commercial homo PPs with an MI of approximately 15 were obtained from S-Oil (HK100, Seoul, Korea; MI = 15, ρ = 0.90 g/mL, $M_{n}$ = 82,392, and $M_{w}$ = 322,152), Lotte Chemical (FR160, Seoul, Korea; MI = 15, ρ = 0.90 g/mL, $M_{n}$ = 91,228, and $M_{w}$ = 314,736), and PolyMirae (HP653P, Seoul, Korea; MI = 16, ρ = 0.90 g/mL, $M_{n}$ = 83,458, and $M_{w}$ = 333,834).

### 2.2. Characterization

#### 2.2.1. Rheological Properties of CR-PP Samples

The melt rheological properties of neat PP and CR-PPs were measured at 200 °C using strain-controlled ARES-G2 (TA Instruments, New Castle, DE, USA) in the cone-and-plate with a diameter of 25 mm and cone angle of 0.1 rad. A frequency sweep test was performed in the frequency range of 0.1 to 300 rad/s at a constant strain of 5%. Disk-shaped PP specimens with a diameter of 25 mm were prepared using a mold to analyze the rheological properties. The mold was heated to 230 °C under a pressure of 5 MPa using a hot press (No. 196 electric heating system test press, Yasuda-Seiki, Hyogo, Japan).

For the fiber-spinning application of homo PPs (HK100, FR160, and HP653P) and CR-PP (with a peroxide content of 200 ppm) for staple fiber production, their rheological properties were measured at 180 °C using MCR-302 (Anton Paar, Graz, Austria) under the abovementioned frequency-sweep mode to be correlated with the melt tension data presented in Section 2.2.6. 

#### 2.2.2. Mechanical Properties of CR-PPs

CR-PP samples for measuring various mechanical properties were manufactured using a family mold in a 130-ton injection machine (SE130EV-A, Sumitomo Heavy Industries, Japan). All samples were aged at room temperature and 50 RH% for 48 h prior to performing the following measurements: MI (ASTM D 1238) at 230 °C and a piston weight of 2.16 kg, tensile strength (ASTM D 638) under 50 mm/min (speed of testing), flexural modulus (ASTM D 790) under 2.5 mm/min (rate of crosshead motion), and Izod impact strength (ASTM D 256) under a 3 J pendulum.

#### 2.2.3. Fourier-Transform Infrared Spectroscopy (FT-IR)

FT-IR (FT-IR4700, Jasco, Tokyo, Japan) was performed to determine the degree of peroxy-promoted oxidation inside CR-PPs. The transmission mode was used in a disk-type PP specimen measuring 10 mm in diameter and 0.2 mm thick prepared using a hot press (No. 196 electric heating system test press, Yasuda-Seiki, Hyogo, Japan) at 230 °C. 

#### 2.2.4. Cold Xylene Soluble (CXS) Property

A CXS (ISO 16152:2005) test was conducted to qualitatively estimate the crystallinity of CR-PPs treated with different peroxide contents. Amorphous atactic parts in PP resin were dissolved in hot xylene liquid (135 °C) under a nitrogen environment and cooled to 20 °C. After filtration through a filter paper, the weight of the remaining crystalline components was measured. 

#### 2.2.5. Volatile Matter Measurement

The weights of the CR-PPs before and after thermal annealing were measured using an electronic scale (ENTRIS2202I-1S, Sartorius Laboratory Instruments GmbH & Co., Göttingen, Germany) to determine the change in the amount of volatile materials (e.g., low-carbon hydrocarbons) inside the sample. The CR-PPs were annealed in an oven at 130 °C for 3 h.

#### 2.2.6. Application to Melt-Spinning Process: Melt Tension and Draw Resonance Instability of PPs

**Figure 2:**
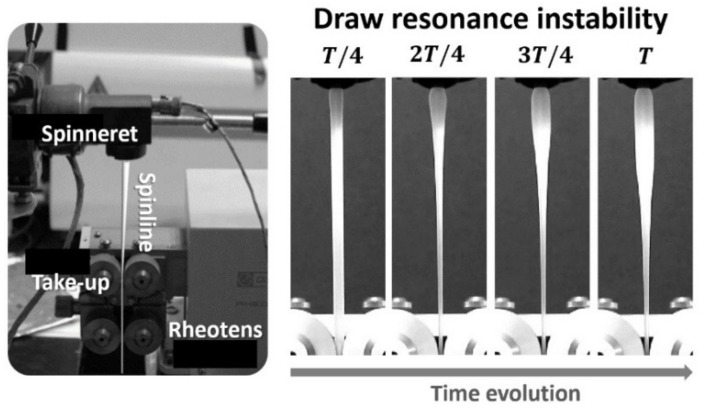
A schematic of melt spinning process and an example of draw resonance instability within one period of oscillation.

The applicability of peroxide-treated CR-PP (with 200 ppm peroxide) to the fiber-spinning process (Figure 2), which is one of representative extensional deformation processes, was confirmed via a comparison with commercial homo PPs (HK100, FR160, and HP653P) as staple fiber products. The melt tensions of PPs in the spinline from the die nozzle to the take-up (25 cm) were measured using a Rheotens (GÖTTFERT, Buchen, Germany) and a single-screw extruder (Collin, Maitenbeth, Germany, D = 30 mm and L/D = 25) under several extrusion rate conditions, i.e., 0.25, 0.5, and 0.75 kg/h at an extrusion temperature of 180 °C. Melt tension measured by gradually increasing take-up speed, which is an important extensional rheological property, is associated with the flow instability in the spinline [36]. One representative flow instability is draw resonance (Figure 2), which is characterized by periodic oscillations of the spinline diameter or the spinline tension over a certain drawdown ratio (i.e., the take-up velocity divided by the inlet velocity at the spinneret or nozzle). The onset of draw resonance instability for the PPs was determined using the drawdown ratio [37,38].

Note that most experiments were repeated three or more times to obtain reliable data.

## 3. Results and Discussion

### 3.1. Effect of Peroxide Content on Physical Properties of CR-PPs via Reactive Extrusion

#### 3.1.1. MI, MWD, and CXS Properties

**Figure 3:**
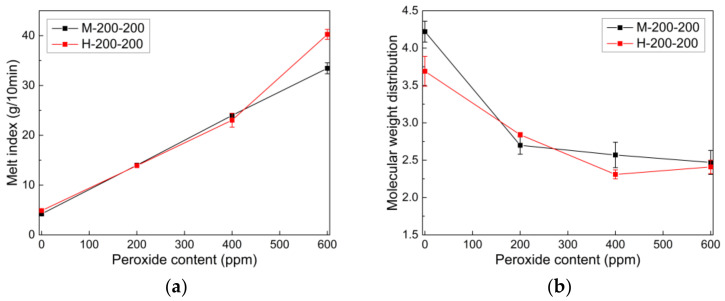

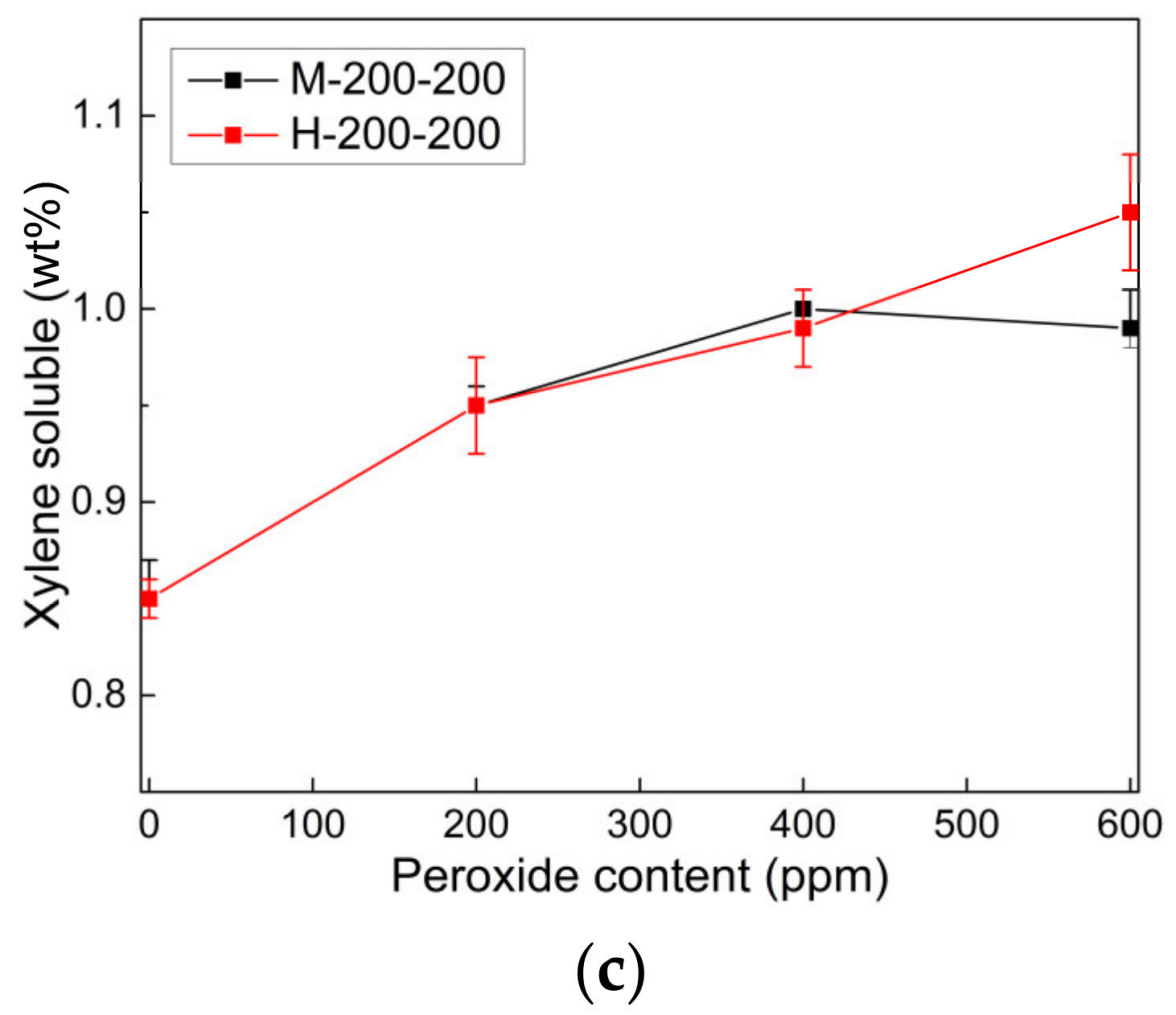
Effect of peroxide content on (**a**) melt index, (**b**) molecular weight distribution (MWD), and (**c**) xylene soluble (CXS) of CR-PPs produced using mild and harsh twin-screw extruders.

Extrusion conditions such as the twin-screw configuration, extruder temperature, and screw speed can significantly affect the chain scission of PP by peroxide inside the twin-screw extruder. First, the effect of peroxide content on various properties (e.g., MI, MWD, and CXS) of PP (HD500) was examined using pilot-scale twin-screw extruders with mild and harsh screw configurations at an extrusion set temperature of 200 °C and screw speed of 200 rpm (Figure 3). Other experimental results obtained at different extrusion temperatures and screw speed are briefly summarized in Section 3.2.2. Some abbreviations of CR-PP samples extruded under different extrusion conditions are designated based on the screw configuration (mild (M) or harsh (H)), extrusion temperature (e.g., 200 °C), and screw speed (e.g., 250 rpm). For example, H-200-250 denotes a CR-PP sample manufactured at 200 °C and 250 rpm using a harsh twin-screw extruder. 

Figure 3a shows the effect of peroxide content on the MI of PPs. The MI tends to increase linearly with the peroxide content, implying that the molecular weight of PP is reduced owing to the more extensive chain scission reaction during the reactive extrusion process. The MI data obtained using mild and harsh screw extruders differed significantly in the CR-PP containing more than 400 ppm of peroxide. In particular, the harsh screws that exerted a higher shear force and a longer residence time inside the extruder resulted in a higher MI.

As the chain scission reaction by peroxide proceeded in the twin-screw extrusion process, the MWD, a key factor in chain length uniformity, varied accordingly. The MWD narrowed gradually as the peroxide content increased (Figure 3b), which is beneficial for enhancing the spinnability of melt-spinning processes by alleviating fiber breakage defects [39]. It is noteworthy that the addition of a small amount of peroxide significantly narrowed the MWD, whereas the MWD of CR-PP extruded via the harsh extrusion method did not differ significantly from that of CR-PP extruded via the mild extrusion method.

**Figure 4:**
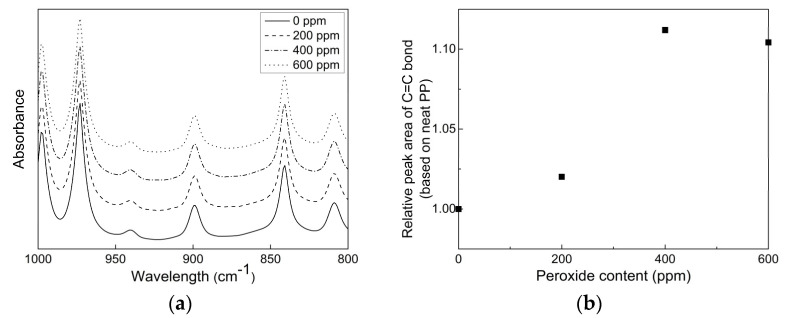
(**a**) FT-IR spectra for CR-PPs with different peroxide contents in the range of 1000 to 800 cm^−^^1^ wavenumber. (**b**) Peak area of C = C bonds for CR-PPs relative to that of neat PP.

Because the amorphous atactic component of semi-crystalline PP dissolved in xylene, the effect of the peroxide content on the xylene-soluble feature in CR-PPs was interpreted as shown in Figure 3c. As the peroxide content increased, the level of CXS increased because unsaturated C = C and C = O bonds formed via chain scission reactions during extrusion suppressed the growth of crystallinity and increased the atactic component inside the CR-PPs. This trend was confirmed by the FT-IR results depicted in Figure 4, where it is indicated that the IR peak at 898 cm^−1^ for C = C bonds [14] increased up to a peroxide content of 400 ppm and then remained above 400 ppm. 

#### 3.1.2. Rheological and Mechanical Properties

**Figure 5:**
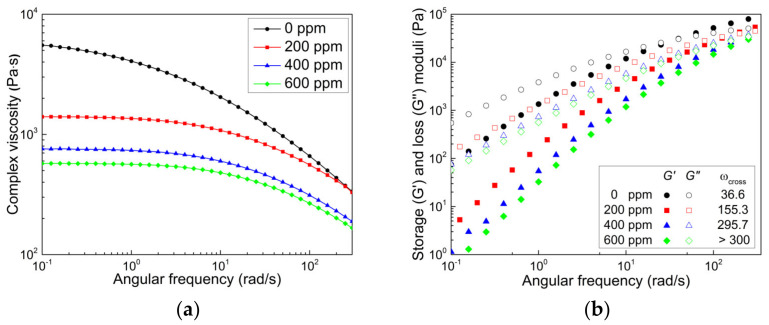
Rheological properties of neat PP and CR-PPs. (**a**) Complex viscosity and (**b**) storage (G’) and loss (G’’) moduli.

The melt rheological properties of neat PP (HD500) and its CR-PPs treated with different amounts of peroxide, i.e., their complex viscosity as well as storage and loss moduli data, were measured at 200 °C (Figure 5). Samples were prepared using a mild twin-screw extruder under the same extrusion conditions as shown in Figure 3. As the amount of peroxide increased, the complex viscosity of PPs decreased, as expected, and the Newtonian regime in the low-intermediate frequency range expanded (Figure 5a). Notably, the addition of a small amount of peroxide (200 ppm) considerably reduced the viscosity level, thereby alleviating non-Newtonian shear thinning. Regarding the viscoelastic modulus data shown in Figure 5b, both the elastic (G’) and viscous (G’’) moduli decreased, and the crossover frequency (ωcross) at the intersection of G’ and G’’ increased with the peroxide content, implying weakened viscoelastic features owing to extensive chain scission. The change in the crossover point directly indicates structural changes in PP, such as the molecular weight and MWD. The higher the peroxide content, the higher the frequency and modulus at the crossover point, supporting that peroxide effectively reduces the molecular weight and narrows the MWD of PP, as shown in Figure 3b and Figure 5a.

A comparison of the various mechanical properties of neat PP and CR-PPs treated with different peroxide contents is shown in Table 2. As the peroxide content increased, the flexural modulus and tensile strength of neat PP and CR-PP decreased slightly, although their properties were not significantly affected by the peroxide content. It is noteworthy that the Izod impact strength was extremely low compared with the other mechanical properties. In the case of general homo PP, lower-molecular-weight polymers exhibit higher stiffness owing to the better orientation of polymer chains and increased growth of crystalline components [40]. In contrast, the mechanical properties of low-molecular-weight CR-PPs after peroxide treatment were not improved because of the formation of unsaturated bonds, which might interfere with crystal formation [41].

### 3.2. Effect of Various Extrusion Conditions on CR-PP Production

#### 3.2.1. Change in Flow Properties inside Twin-Screw Extruder

**Figure 6:**
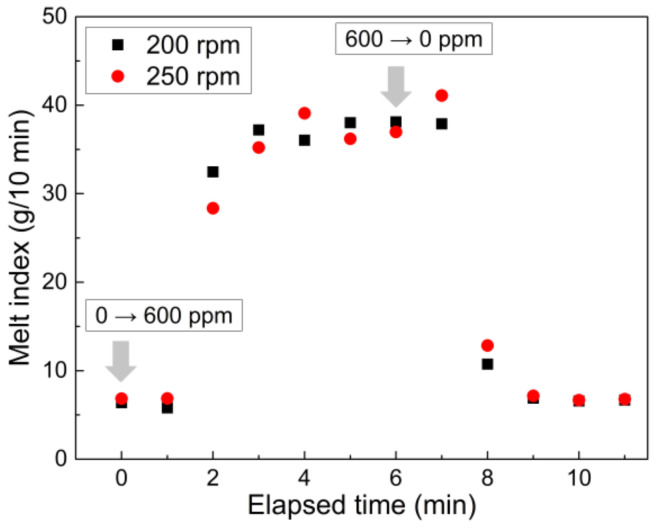
Real-time variation of melt index after step changes of peroxide content in input polymer materials (0 → 600 ppm and 600 → 0 ppm) under extrusion conditions of 215 °C as well as 200 and 250 rpm.

During the continuous pilot-scale reactive extrusion of PP, residues inside the extruder can affect subsequent products containing different peroxide contents. When the peroxide content for CR-PP production was changed stepwise from 0 to 600 ppm (and from 600 ppm to 0) in the hopper during the continuous harsh twin-screw extrusion under 200 and 250 rpm conditions and a temperature of 215 °C, the MI of the CR-PP extrudates changed gradually during the transit period, based on the residence time of melt polymers traveling between twin-screws inside the extruder (Figure 6). After changing the peroxide content in the hopper to 600 ppm, 1 min was required to fully apply peroxide to the extruder. Thereafter, the MI increased during the residence time of approximately 3 min, and conversely, changing the peroxide content to 0 ppm decreased the MI up to an elapsed time of 3 min, as in the previous case. Note that screw speed did not significantly alter the MI of the PP extrudates.

**Figure 7:**
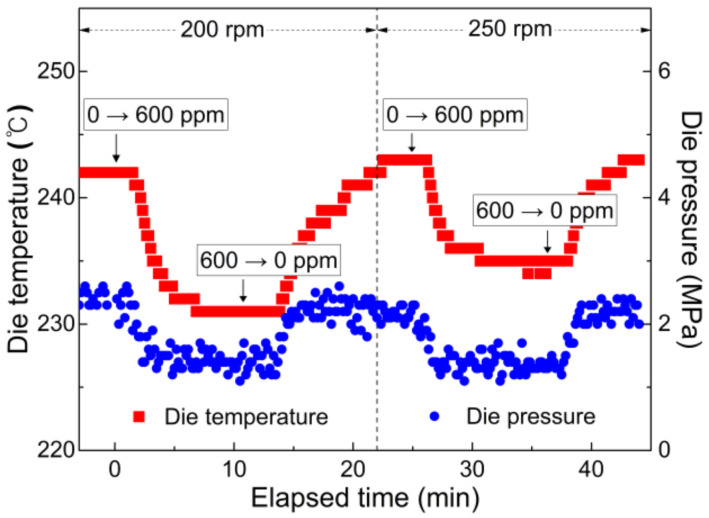
Variation in temperature and pressure at die position after step changes of peroxide content in input polymer materials (0 → 600 ppm and 600 → 0 ppm) under extrusion conditions of 215 °C as well as 200 and 250 rpm.

Additionally, changes in the temperature and pressure at the die were monitored in real time after changing the peroxide content in the hopper or screw speed (Figure 7) at an extrusion set temperature of 215 °C. The initial die temperature for neat PP (approximately 241 °C) was considerably higher than the set temperature because of the viscous dissipation effect. Similar to the previous procedure, the die temperature and pressure started to decrease during the transition period after the peroxide content in the hopper was changed from 0 to 600 ppm. This temperature decrease is ascribed to weakened viscous dissipation. After a transition period of 4–5 min, CR-PP containing 600 ppm of peroxide was stably extruded at a relatively low die temperature of 232 °C. When neat PP (HD500) without peroxide (0 ppm) was introduced into the hopper, the die temperature increased noticeably during the transition period. When the screw speed was further increased to 250 rpm, the die temperature increased to 244 °C owing to the higher shear rate and stronger viscous dissipation. The pattern of the increasing–decreasing die temperature at 250 rpm was the same as that in the previous 200 rpm case. The temporal change in the die pressure was analogous to the change in die temperature with respect to the peroxide content and screw speed conditions, albeit with slight fluctuations.

#### 3.2.2. Change in MI, MWD, CXS, and Volatile Materials

**Figure 8:**
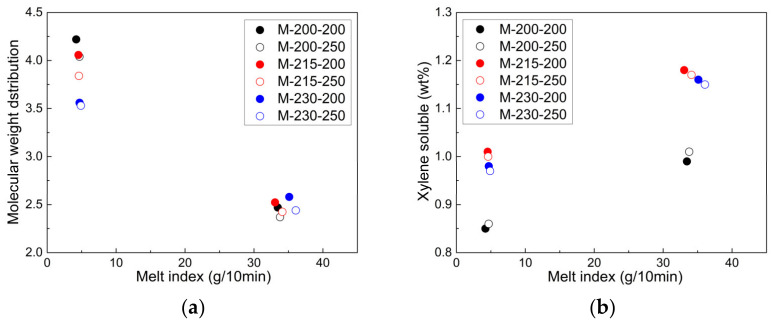

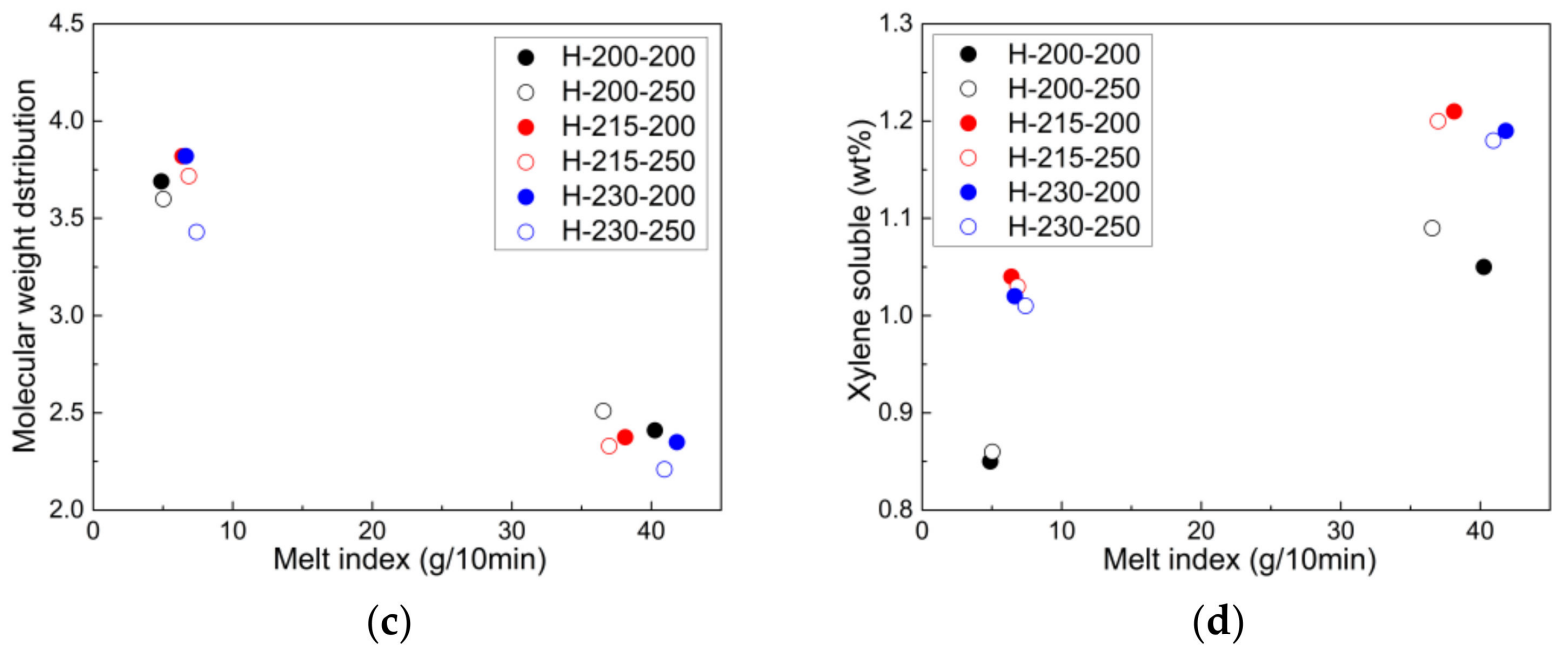
Relationship of (**a**,**c**) molecular weight distribution and (**b**,**d**) xylene-soluble property with respect to melt index of CR-PPs under various extrusion conditions.

Figure 8 shows a comparison of the changes in the MWD and CXS for neat PP (HD500) and CR-PP (with 600 ppm of peroxide) under different extrusion conditions, such as mild and harsh twin-screws, the extrusion temperature, and the screw speed. In the x-axis of the figures, the data at approximately MI = 5 and MI = 35 represent those for neat PP and CR-PP, respectively. Compared with mild twin-screw extrusion, harsh extrusion rendered the MI and CXS of PPs a little higher, resulting in a narrower MWD owing to the more active chain scission reaction. Furthermore, it is evident that the screw speed (200 and 250 rpm) more affected the MIs of the PP samples extruded via the harsh twin-screw extruder, in contrast to the mild extrusion. A high extrusion temperature increased the CXS value in the CR-PPs by generating more unsaturated bonds. The optimal peroxide content for manufacturing CR-PP resin should be determined meticulously by balancing the required structural and physical properties.

**Figure 9:**
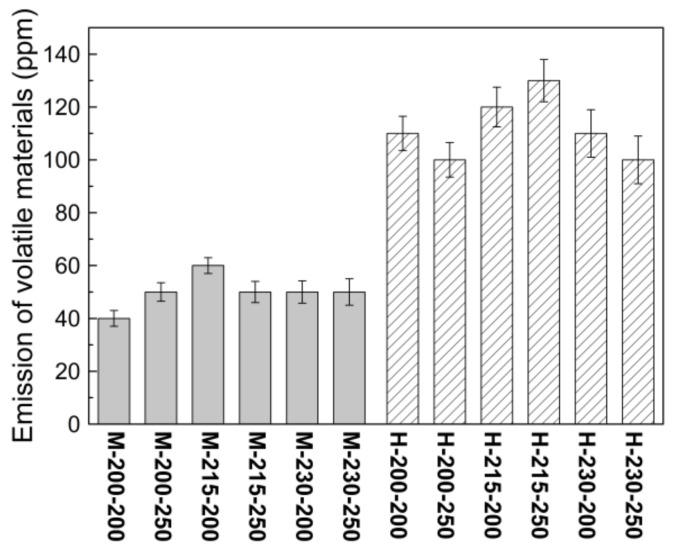
Comparison of amounts of evaporated volatile materials in extruded CR-PP samples under various extrusion conditions.

The emission of volatile materials, such as low-carbon hydrocarbons, inside reactive-extruded thermoplastic polymers depends on the chemical structures of the polymers and the processing conditions. Based on issues pertaining to environmental regulations and the operating situations, the amounts of volatile materials inside CR-PP samples extruded via mild and harsh twin-screw extruders at different extrusion temperatures (200 °C, 215 °C, and 230 °C) and screw speed (200 and 250 rpm) were measured using the experimental method presented in Section 2.2.5 (Figure 9). The amount of volatile materials emitted from the extruded CR-PPs was affected more by the screw configuration (mild or harsh) than by other extrusion conditions. Polymer decomposition was more active inside the harsh twin-screw extruder, which was attributed to a relatively high shear force and a long residence time.

### 3.3. Application of CR-PP in Fiber-Spinning Process: Comparison of Melt Tension and Draw Resonance Instability

**Figure 10:**
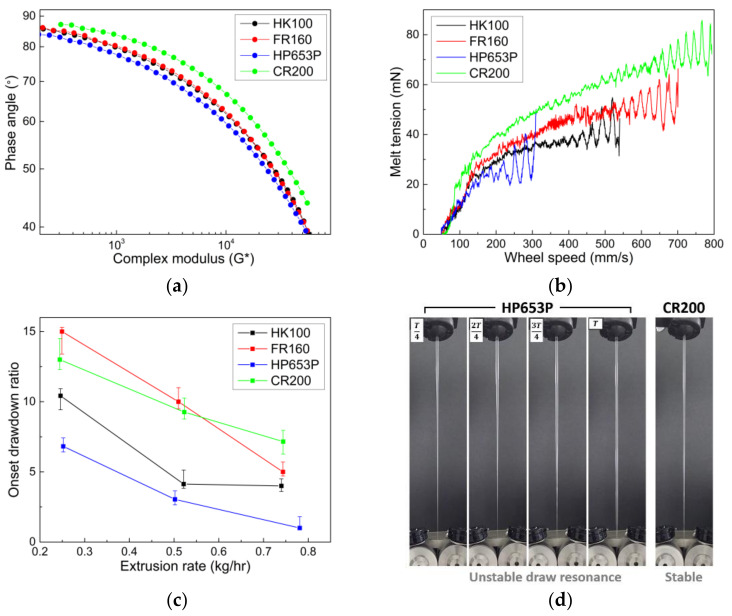
Comparison of (**a**) van Gurp–Palmen plot, (**b**) melt tension, and (**c**) draw resonance onset of CR-PP (CR200) and homo PPs (HK100, FR160, and HP653P) in melt-spinning process. (**d**) Fiber formations from the spinneret to take-up for HP653P (unstable state) and CR200 (stable state) under conditions of drawdown ratio of 8 and extrusion rate of 0.5 kg/h. T is the period of oscillation.

As illustrated in Figure 2, melt spinning process is a useful method to manufacture highly oriented and uniaxially deformed fibers along the spinline from the spinneret to take-up. In the spinning experiments, the melt polymer was stretched along the spinline by drawing at the take-up of Rheotens. To verify the applicability of CR-PP in the melt spinning process, four samples with similar MI values of approximately 15 were selected: CR-PP produced using 200 ppm peroxide (CR200, MI = 14.5, ρ = 0.90 g/mL, $M_{n}$ = 108,830, and $M_{w}$ = 308,344) and homo PPs (HK100, FR160, and HP653P). For similar MIs, the CR-PP showed a smaller molecular weight and narrow MWD compared with the homo PPs. First, the viscoelastic properties of PP samples measured at 180 °C were compared, as shown by the van Gurp–Palmen plots for phase angle ($=\tan^{-1}\frac{G^{\prime \prime }}{G^{\prime }}$) vs. complex modulus ($G^{*}=\sqrt{{\left(G^{\prime }\right)}^{2}+{\left(G^{\prime \prime }\right)}^{2}}$) in Figure 10a, based on their G’ and G’’ data. The elasticity of CR200 was relatively low in comparison with that of the other homo PPs, which is beneficial for stabilizing the fiber-spinning process.

By employing the lab-scale single-screw extruder and Rheotens equipment, CR200 and three homo PPs were applied to the fiber-spinning process. In the Rheotens test, melt tension was defined as the force to draw the melt strand in the spinline from the spinneret to the take-up roll, and it can be expressed as a function of the take-up velocity. Melt tensions for PPs were measured in the Rheotens by gradually increasing the take-up speed (or drawdown ratio). The extrusion temperature was set at 180 °C. As shown in Figure 10b, the melt tension generally increased with the take-up speed and fluctuated significantly over the draw resonance onset for all the PP cases. It was demonstrated that the CR200 sample exhibited superior spinnability compared with the other PPs because its melt tension in the spinline was significantly higher and it fluctuated at higher take-up speeds. In general, a higher melt tension enlarges the stable operating region during polymer extensional deformation processes [42].

Above the critical take-up speed (or drawdown ratio), draw resonance instability occurs in the spinline, exhibiting periodic variations in the melt tension and spinline diameter, which is detrimental to the production of uniform fiber products. Based on the melt tension data and direct observation of the variation in the spinline diameter along the take-up speed, draw resonance onsets for the PP samples were determined by changing the extrusion rate, as shown in Figure 10c. The spinnability of the CR200 was similar to that of the FR160 sample and superior to that of the other two samples. It is well known that extensional-thinning polymers, such as PP and high density polyethylene (HDPE), with linear chain structures render the spinning system less stable as the viscoelasticity or Deborah number increases [37,38]. Hence, the less elastic CR200 exhibited a more stabilizing effect than the other PPs. Figure 10d shows different fiber formations in the spinline for HP653P and CR200 samples under spinning conditions of drawdown ratio of 8 and extrusion rate of about 0.5 kg/h. The HP653P is in the unstable draw resonance state, which exhibits periodic oscillations of spinline diameter within one period of oscillation, whereas the CR200 is in a stable state. In summary, CR-PP is expected to offer promising applications in the melt-spinning process, considering its desirable properties of narrow MWD, low elasticity, and high melt tension.

## 4. Conclusions

Controlled rheology is a useful method for generating more uniform polymer chains via the addition of peroxides during reactive extrusion. In this study, the role of peroxide content in altering the structural and rheological properties of CR-PPs was investigated via chain scission reactions inside pilot-scale twin-screw extruders. Small amounts of peroxide up to 600 ppm effectively increased the MI, thereby narrowing the MWD of CR-PPs. The chain cleavage of PP by peroxide increased the amount of atactic moieties, which prevented crystallization via the formation of unsaturated chain ends such as C = C. The effect of extrusion conditions, such as the screw configuration, extruder temperature, and screw speed, on the properties of CR-PPs was examined. At extrusion temperatures above 215 °C, the harsh twin-screw extruder, which provided a high shear force, indicated a distinct peroxide effect as compared with the mild twin screw extruder. By considering the residence time variation of the peroxide remaining inside the extruder, it was confirmed that the extrusion process stably reached a new steady state within a few minutes, based on the stepwise change of the input materials with different peroxide contents. Meanwhile, by comparing the melt tension exerting on the spinline and the onset of draw resonance instability in the melt-spinning process for CR-PP and three commercially available homo PPs, it was discovered that CR-PP with a relatively lower elasticity and higher melt tension provided a more stable spinnability region in the melt-spinning process. Hence, CR-PPs with target-oriented properties are industrially suitable for producing uniform staple fibers and spunbonds through high-speed spinning processes.

## Figures and Tables

**Table 1:** Temperature conditions inside two twin-screw (mild and harsh) extruders depicted in Figure 1. Extrusion rate is 50 kg/h and screw speed is 200 or 250 rpm.

	Mild Extrusion			Harsh Extrusion		
Set Temp. (°C)	200	215	230	200	215	230
Zone 1	160	160	160	160	160	160
Zone 2	175	175	175	175	175	175
Zone 3	190	190	190	190	190	190
Zone 4	190	200	210	190	190	200
Zone 5	200	200	210	190	200	210
Zone 6	200	215	230	190	200	210
Zone 7	200	215	230	200	215	230
Zone 8	200	215	230	200	215	230
Zone 9	200	215	230	200	215	230
Zone 10	-			200	215	230
Zone 11				200	215	230
Zone 12				200	215	230
Zone 13				200	215	230

**Table 2:** Effect of peroxide content on mechanical properties of PPs such as flexural modulus, tensile strength, and Izod impact strength.

Peroxide Content (ppm)	Flexural Modulus (MPa)	Tensile Strength (MPa)	Izod Impact Strength (J/m)
0	1520 ± 10	34.3 ± 0.5	13.3 ± 0.7
200	1516 ± 4	33.6 ± 0.1	15.2 ± 1.0
400	1509 ± 10	33.4 ± 1.0	15.7 ± 0.7
600	1500 ± 10	33.3 ± 0.2	16.3 ± 0.3

## Data Availability

Not applicable.
